# Surgical Resection with Posterior Rectus Sheath Preservation and Mesh Reconstruction without Pursuing Wide Negative Margins for an Abdominal Wall Desmoid in Familial Adenomatous Polyposis: A Case Report

**DOI:** 10.70352/scrj.cr.25-0584

**Published:** 2025-12-26

**Authors:** Makoto Hasegawa, Tomoyuki Momma, Erina Suzuki, Yuko Hashimoto, Hiroki Yago, Misato Ito, Takuro Matsumoto, Daisuke Ujiie, Shun Chida, Hirokazu Okayama, Motonobu Saito, Wataru Sakamoto, Koji Kono

**Affiliations:** 1 Department of Gastrointestinal Tract Surgery, Fukushima Medical University School of Medicine, Fukushima, Fukushima, Japan; 2 Department of Gastroenterology, Kita Fukushima Medical Center, Date, Fukushima, Japan; 3 Department of Diagnostic Pathology, Fukushima Medical University School of Medicine, Fukushima, Fukushima, Japan; 4 Department of Gastroenterological Surgery, Aizu Medical Center, Fukushima Medical University, Aizu, Fukushima, Japan

**Keywords:** familial adenomatous polyposis, abdominal wall desmoid, function-sparing abdominal wall surgery

## Abstract

**INTRODUCTION:**

Desmoid tumors are locally aggressive, non-metastatic neoplasms that develop in up to 20% of patients with familial adenomatous polyposis (FAP). While active surveillance is the initial approach, surgery may be indicated for symptomatic or progressive disease; however, the optimal surgical strategy remains debated.

**CASE PRESENTATION:**

We present the case of a 48-year-old man with FAP and a history of two previous laparotomies. He developed a progressive and symptomatic abdominal wall desmoid tumor refractory to non-operative management, including medical therapy. He underwent a surgical resection without pursuing wide negative margins. Intraoperatively, the tumor was adherent to the prior midline incision scar and anterior rectus sheath. After resection, the resulting 100 × 50 mm fascial defect was repaired with a synthetic mesh. Histopathology confirmed the desmoid tumor with microscopically negative (R0) resection margins, and no evidence of recurrence was observed at the 9-month follow-up.

**CONCLUSIONS:**

Surgical resection without pursuing wide negative margins, previously described in sporadic desmoids, may be considered a feasible option for selected FAP-associated abdominal wall tumors, balancing local control with no apparent postoperative abdominal wall functional deficit.

## Abbreviations

ERCP
: endoscopic retrograde cholangiopancreatography
ESD
: endoscopic submucosal dissection
FAP
: familial adenomatous polyposis
LECS
: laparoscopy and endoscopy cooperative surgery
NSAID
: non-steroidal anti-inflammatory drug

## INTRODUCTION

Desmoid-type fibromatosis (hereafter referred to as desmoid tumor) is a rare, locally aggressive fibroblastic neoplasm characterized by infiltrative growth and a lack of metastatic potential.^1)^ Desmoid tumors are predominantly sporadic, with an estimated annual incidence of 2.4–4.3 per million people, accounting for approximately 0.03% of all neoplasms and fewer than 3% of soft tissue tumors.^1)^

A subset of desmoid tumors is associated with FAP, comprising approximately 7.5%–16% of all cases. Among patients with FAP, desmoid tumors develop in approximately 8%–20% of individuals.^2,3)^ In FAP-associated cases, tumor locations include intra-abdominal (53%), abdominal wall (24%), and extremities (9%), with the remainder being unknown or multiple sites.^2)^ Identified risk factors for desmoid tumor development include prior abdominal surgery, an APC gene mutation at codon 1444, and a family history of desmoid tumors.^2)^

Historically, surgical resection was considered the first-line treatment. However, treatment strategies have changed drastically over the past decades.^4)^ Currently, active surveillance is recommended for asymptomatic cases, while surgery is typically reserved for symptomatic or progressive disease, particularly when the tumor causes pain, functional impairment, or threatens vital structures.^4)^

There is ongoing debate regarding the optimal surgical margin. Some advocate for achieving a microscopically negative (R0) resection,^5)^ whereas others report there is no significant difference in recurrence rates even with a microscopically positive (R1) margin.^6,7)^ We herein report the case of an FAP-associated abdominal wall desmoid tumor successfully managed with a less-invasive surgery that preserved the posterior rectus sheath.

## CASE PRESENTATION

The patient was a 48-year-old man with a history of FAP. His surgical history included a subtotal colectomy at age 24 and a partial duodenal resection at age 46 for duodenal adenoma. The latter procedure, attempted via LECS, required conversion to open laparotomy due to intraoperative perforation. He also had recurrent adhesive small-bowel obstruction.

**Fig. 1:**
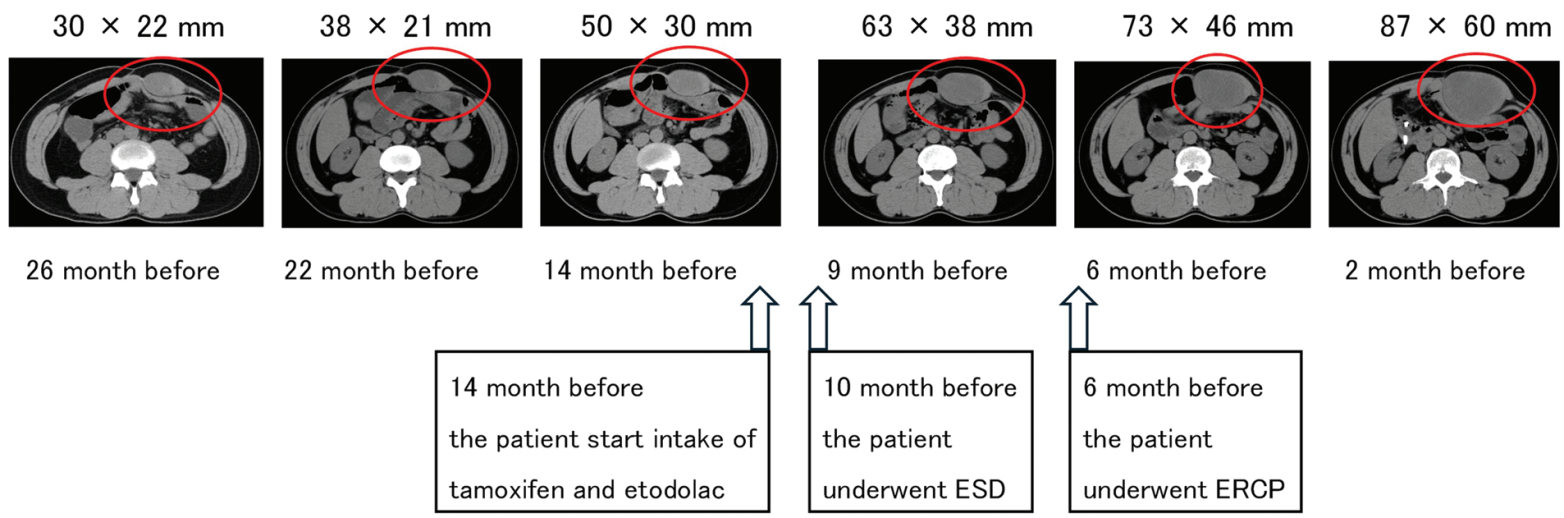
Serial abdominal CT images illustrating progressive enlargement of the abdominal wall desmoid tumor (red circles). Pharmacotherapy with tamoxifen and etodolac was initiated 14 months before surgery; ESD for a duodenal adenoma was performed 10 months before surgery; and ERCP for a gallbladder stone was performed 6 months before surgery. ERCP, endoscopic retrograde cholangiopancreatography; ESD, endoscopic submucosal dissection

At 2 years and 2 months before the present operation, abdominal CT incidentally revealed a left upper-quadrant abdominal-wall mass that was diagnosed as a desmoid tumor, and active surveillance was initiated. CT performed 14 months preoperatively showed progressive enlargement, leading to the commencement of tamoxifen and oral etodolac, an NSAID. Despite this medical therapy, the tumor continued to grow and became symptomatic with pain, ultimately reaching a size of 87 mm in the axial image (**Fig. 1**). During this period, 10 months before surgery, the patient also underwent ESD for a duodenal adenoma. Additionally, 6 months before surgery, ERCP was performed for a gallbladder stone. Due to the tumor’s progression and symptoms, he subsequently underwent a less invasive resection, which prioritized function over achieving wide, microscopically negative (R0) margins.

**Fig. 2:**
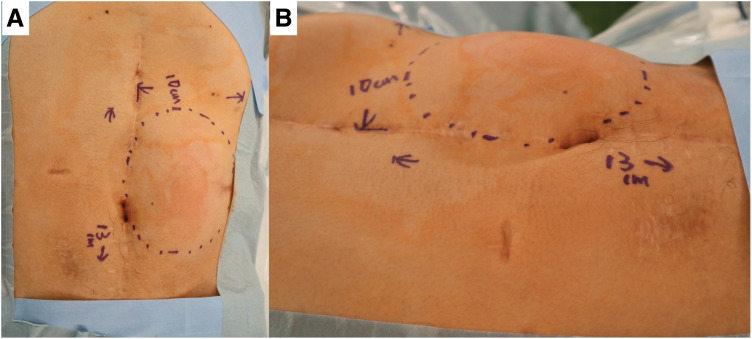
The tumor, measuring 130 × 100 mm, was located within the abdominal wall left upper quadrant. (**A**) anterior view. (**B**) lateral view.

**Fig. 3:**
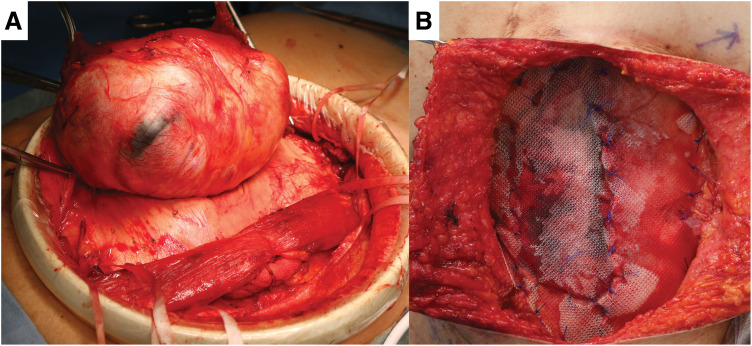
Intraoperative findings of the tumor resection and abdominal wall reconstruction. (**A**) The tumor was resected from the rectus abdominis muscle, with careful preservation of the posterior rectus sheath. (**B**) The resulting 100 × 50 mm anterior sheath defect was reconstructed using a 150 × 150 mm Bard Mesh.

The procedure was performed with the patient in the supine position under general anesthesia with epidural analgesia. On physical examination, a tumor measuring approximately 130 × 100 mm was palpated in the upper left quadrant of the abdominal wall (**Fig. 2**). A longitudinal skin incision was made overlying the tumor. The anterior rectus sheath was incised to expose the rectus abdominis muscle, which was found to contain the tumor. An Alexis O Wound Protector/Retractor (Applied Medical Resources, Rancho Santa Margarita, CA, USA) was applied to maintain the surgical field. The tumor was firmly adherent to the previous midline incision scar and partially to the anterior sheath. The portion of the rectus abdominis muscle involved by the tumor was resected. The tumor was then carefully dissected from the medial scar tissue and excised (**Fig. 3A**). After excising the tumor, we attempted primary closure of the anterior rectus sheath; however, a residual 100 × 50 mm fascial defect remained at the resection site. The defect was reconstructed using a 150 × 150 mm Bard Mesh (BD, Franklin Lakes, NJ, USA) (**Fig. 3B**). Additionally, Seprafilm (KAKEN Pharmaceutical, Tokyo, Japan) was applied to the posterior aspect of the mesh to prevent adhesion to the posterior sheath. The skin was closed with dermal sutures, and the operation was concluded. The operative time was 254 minutes, and intraoperative blood loss was 30 mL.

The patient developed postoperative ileus. After an unsuccessful attempt to start a diet on POD 1, a nasogastric tube was placed on POD 2 for nausea. This was followed by the insertion of a Transnasal Intestinal Decompression Tube on POD 6, which was removed on POD 10. Oral intake was then resumed, and the patient was discharged on POD 15.

**Fig. 4:**
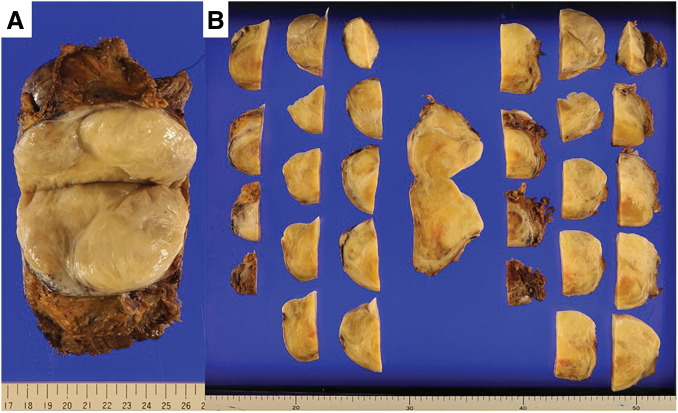
The resected tumor measured 120 × 90 × 65 mm and weighed 492 g. (**A**) Bisected specimen showing the cut surface; (**B**) serially sectioned specimen.

**Fig. 5:**
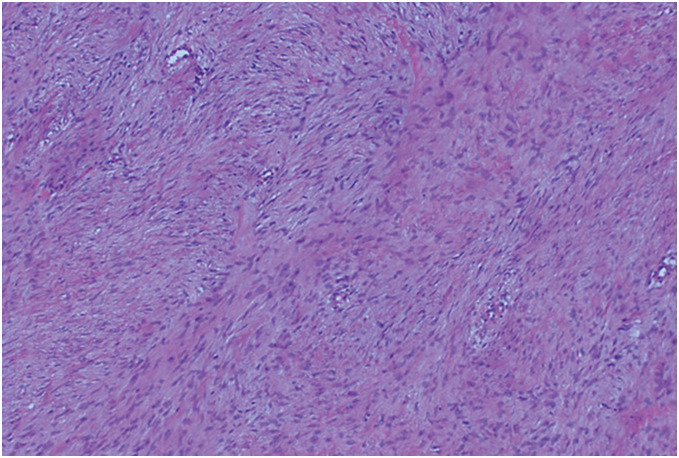
Hematoxylin and eosin staining (100x magnification): Histology showed a tumor consisting of spindle cells arranged in fascicles.

The resected tumor measured 120 × 90 × 65 mm and weighed 492 g (**Fig. 4**). Histology showed a tumor consisting of spindle cells arranged in fascicles (**Fig. 5**). Immunohistochemical staining revealed positivity for β-catenin, CD34, and Desmin, and negativity for pan-cytokeratin (AE1/AE3), EMA, and S-100 protein. The mitotic activity was noted at a rate of up to 7 per 50 high-power fields. Pathological examination confirmed the desmoid tumor with R0 margins. At the 9-month follow-up, there was no evidence of recurrence. The patient’s preoperative abdominal pain had resolved, and he reported no restrictions in daily physical activities.

## DISCUSSION

We have presented the case of a progressive, symptomatic, FAP-associated abdominal wall desmoid tumor successfully managed with surgical resection without pursuing wide negative margins. This case is significant as it supports the application of this less-invasive technique to the FAP-associated subtype, a population for whom the optimal surgical approach remains debated and evidence is limited.^8)^

The management of desmoid tumors, including those associated with FAP, has evolved significantly. The current treatment paradigm favors a multidisciplinary approach, starting with active surveillance and progressing to medical therapies.^3,4)^ Surgery is typically reserved for cases that are refractory to conservative management.^3,4)^ According to a previously published systematic review, stable disease was the most common outcome in AS for abdominal desmoid (median, 59%; IQR, 37%–69%), while smaller proportions of patients experienced either tumor progression (median, 20%; IQR, 13%–35%) or some form of regression (median, 19%; IQR, 3%–23%).^9)^ Our patient’s clinical course aligns perfectly with this modern strategy; surgery was indicated only after both active surveillance and pharmacotherapy—including NSAIDs and tamoxifen—failed to control tumor growth or alleviate symptoms.^3)^

The key surgical principle in our case was deliberately foregoing the pursuit of wide negative margins. This approach has been described as a feasible option for sporadic (non-FAP-related) abdominal wall desmoids.^8)^ A previous study evaluating this technique in 15 patients with sporadic abdominal wall desmoids reported a low recurrence rate (6.7%, 1/15) over a median follow-up of 38 months, with minimal need for reconstruction (13%, 2/15; fascia lata patch).^8)^ Our case extends this principle to an FAP-associated desmoid, suggesting that a similar function-sparing philosophy is applicable. However, due to our patient’s tumor size and dense adhesions, a fascial defect remained after resection that could not be closed primarily, necessitating reconstruction.

In our case, the resulting fascial defect required reconstruction with a synthetic mesh. This outcome highlights a crucial point: while the philosophy of foregoing wide negative margins aims to minimize morbidity, the specific characteristics of the tumor, such as its size and adherence, may still necessitate prosthetic reinforcement. Nevertheless, this strategy successfully avoided the need for more extensive and morbid procedures, such as reconstruction with a muscle flap or by posterior component separation, which are often required to close large defects created by traditional wide resections.^10,11)^ Furthermore, avoiding an overly aggressive resection is supported by the current understanding of desmoid biology, as an excessive margin can be counterproductive. Desmoid tumor recurrence can happen not only from a microscopically positive margin but also as a de novo tumor developing within the surgical field, triggered by the trauma of the resection itself.^3,12)^ Therefore, our approach—which achieved a complete resection while preserving surrounding healthy tissue—aligns with the modern goal of balancing adequate local control with the preservation of abdominal wall integrity.

While FAP-related desmoids differ from sporadic desmoids—often having a greater recurrence rate^13)^—we suggest that the philosophy of foregoing wide margins is particularly suitable for this population. Surgical trauma is a well-established trigger for FAP-associated desmoids, with most developing at prior surgical sites within 5 years of an operation.^13,14)^ An aggressive, wide-margin resection itself constitutes significant surgical trauma, which may paradoxically trigger a “second hit” and promote new tumor growth or recurrence.^13)^ The progressive growth of the tumor in our patient is likely attributable to his extensive history of major abdominal surgeries, including a subtotal colectomy and, in particular, a partial duodenal resection. Regarding ESD and ERCP, no previous studies have linked these procedures to desmoid tumor progression. In addition, tumor enlargement was already evident when the ESD and ERCP were performed; therefore, their contribution in this case remains uncertain.

Postoperative ileus occurred in this patient and is likely multifactorial, related to surgical and anesthetic factors. Importantly, there was no intra-abdominal manipulation in this case. Postoperative ileus has been reported even with surgical procedures that do not reach the abdominal cavity, such as Extended View Totally Extraperitoneal Repair,^15)^ and non-abdominal procedures like spinal surgery.^16)^ These events are thought to reflect opioid-induced hypomotility, autonomic dysregulation related to surgical stress, and inflammatory mediators, even in the absence of direct bowel manipulation.^16)^ In addition, a history of prior abdominal surgery is a recognized risk factor for postoperative ileus.^17)^ In our case, this risk was likely compounded by a heightened susceptibility to gastrointestinal dysmotility in a patient with a history of multiple prior laparotomies and adhesive small-bowel obstruction.

This case report has several limitations. First, as a single case study, its findings lack generalizability. Second, formal, validated instruments were not used to quantitatively evaluate either QOL or postoperative functional outcomes. Finally, the 9-month postoperative follow-up period is brief, particularly for assessing long-term recurrence.

## CONCLUSIONS

This case demonstrates that surgical resection without pursuing wide negative margins can be a safe and effective option for carefully selected patients with symptomatic FAP-associated abdominal wall desmoids that are refractory to conservative management. It successfully balances the need for local tumor control with no apparent postoperative abdominal wall functional deficit. Further accumulation of cases and longer-term follow-up are necessary to validate these findings and establish the role of this technique in the standard management algorithm for this challenging condition.

## DECLARATIONS

### Availability of data and materials

All data generated or analyzed during this study are included in this published article.
